# Air Pollution Drives Macrophage Senescence through a Phagolysosome–15-Lipoxygenase Pathway

**DOI:** 10.4049/immunohorizons.2300096

**Published:** 2024-04-16

**Authors:** Sarah A. Thomas, Hwan Mee Yong, Ana M. Rule, Naina Gour, Stephane Lajoie

**Affiliations:** * W. Harry Feinstone Department of Molecular Microbiology and Immunology, Johns Hopkins Bloomberg School of Public Health, Baltimore, MD; † Department of Environmental Health and Engineering, Johns Hopkins Bloomberg School of Public Health, Baltimore, MD; ‡ Solomon H. Snyder Department of Neuroscience, Johns Hopkins School of Medicine, Baltimore, MD; § Department of Otolaryngology, Johns Hopkins School of Medicine, Baltimore, MD

## Abstract

Urban particulate matter (PM; uPM) poses significant health risks, particularly to the respiratory system. Fine particles, such as PM2.5, can penetrate deep into the lungs and exacerbate a range of health problems, including emphysema, asthma, and lung cancer. PM exposure is also linked to extrapulmonary disorders such as heart and neurodegenerative diseases. Moreover, prolonged exposure to elevated PM levels can reduce overall life expectancy. Senescence is a dysfunctional cell state typically associated with age but can also be precipitated by environmental stressors. This study aimed to determine whether uPM could drive senescence in macrophages, an essential cell type involved in particulate phagocytosis-mediated clearance. Although it is known that uPM exposure impairs immune function, this deficit is multifaceted and incompletely understood, partly because of the use of particulates such as diesel exhaust particles as a surrogate for true uPM. uPM was collected from several locations in the United States, including Baltimore, Houston, and Phoenix. Bone marrow–derived macrophages were stimulated with uPM or reference particulates (e.g., diesel exhaust particles) to assess senescence-related parameters. We report that uPM-exposed bone marrow–derived macrophages adopt a senescent phenotype characterized by increased IL-1α secretion, senescence-associated β-galactosidase activity, and diminished proliferation. Exposure to allergens failed to elicit such a response, supporting a distinction between different types of environmental exposure. uPM-induced senescence was independent of key macrophage activation pathways, specifically inflammasome and scavenger receptors. However, inhibition of the phagolysosome pathway abrogated senescence markers, supporting this phenotype’s attribution to uPM phagocytosis. These data suggest that uPM exposure leads to macrophage senescence, which may contribute to immunopathology.

## Introduction

Urban particulate matter (uPM) pollution significantly threatens human health. The American Lung Association’s annual State of the Air 2023 report suggests that one third of Americans live in areas with a failing grade for air pollution (1). Globally, it has been estimated that fossil fuel–derived PM2.5 is responsible for as many as 10 million deaths annually (2), and increasing air pollution is estimated to affect half of the world’s population (3). Fossil fuels contribute to PM directly and indirectly through the ecological effects of climate change. In particular, the contribution of wildfire smoke to PM2.5 levels has recently been highlighted as a key obstacle to improving air quality in the United States (4). The association between PM exposure and poor health outcomes is well established, with multisystem implications including respiratory, neurological, and cardiac sequelae (1, 5). Notably, even acute uPM exposure is sufficient to cause harm and has been associated with an increased risk for myocardial infarction (6).

Although uPM exposure is associated with immune dysfunction (7–9), the molecular mechanisms driving such dysfunction are incompletely understood. Macrophages are highly phagocytic immune cells and are the primary cells responsible for responding to environmental exposures (10). Because macrophages are known to regulate the tone and magnitude of immune responses, dysregulated functionality can have myriad downstream consequences. PM exposure has been linked to senescence in various cell types, including fibroblasts (11); this association has yet to be explored in the context of macrophages. Previous investigations have highlighted the impacts of PM exposure on cell functionality in the context of infection (12). This includes PM-mediated deficits in the innate immune function of epithelial cells attributed to senescence (13). Moreover, senescence is usually considered in the context of aging but can also be precipitated by environmental stressors, known as disease-related senescence (14). Given the suboptimal functionality of senescent cells, which no longer undergo cell division, macrophage senescence may contribute to the immune dysfunction observed in response to PM exposure.

The consequences of senescence vary considerably according to the initiating event and affected cell type. Manifestations of senescence include cell-cycle arrest, lysosome dysfunction, accumulation of oxidative stress, and the development of DNA damage (14). The pathogenic potential of senescent macrophages is highlighted in a recent report that the depletion of these cells improves outcomes in a KRAS-driven lung cancer model (15). Beyond the lungs, senescent macrophages also have been implicated in muscular dystrophy (16) and age-related adipose tissue dysfunction (17). The phenotypic heterogeneity of senescent cells necessitates concurrently evaluating multiple hallmarks of senescence, including cell-cycle arrest, enhanced lysosomal mass and activity, lipid accumulation, and an aberrant secretory phenotype (18).

In this study, we sought to evaluate the possibility that senescence is an outcome of uPM exposure in macrophages. Although diesel exhaust particle (DEP) is often used as a surrogate for PM (19), this may be oversimplified because uPM is highly heterogeneous. Its composition varies according to city of origin and season (20). Moreover, we have previously demonstrated that airway exposure of mice to uPM triggers a lung inflammatory response not observed in response to simpler particulates, such as DEP or coal fly ash (CFA) (21). In addition, monosodium urate (MSU) and nanosized silica oxide (nano-SiO_2_) were included as homogenous (single-constituent) particulate controls, previously demonstrated to induce inflammatory cell responses (22–24). To this end, we used PM collected from several urban locations and evaluated key metrics of cellular senescence. PM samples were chosen to represent various urban locations in the continental United States. In addition, because PM is part of the exposome along with other exposures, such as allergens, we wanted to ascertain whether there were unique biological processes elicited by uPM compared with various noninfectious respiratory exposures. To this end, we chose predominant sources of allergens: house dust mite (HDM) and ragweed extracts. Unlike with sources of allergens (HDM and ragweed), exposure of bone marrow–derived macrophages (BMDMs) to uPM results in the acquisition of a senescent profile. This offers insight into a potential mechanism by which uPM-mediated immune dysfunction may arise.

## Materials and Methods

### Sources of PM

MSU and nano-SiO_2_ were obtained from Invivogen. Characteristics of urban PMs are compiled in Table I. DEP, also known as Standard Reference Material (SRM) 1650b, and urban particulate SRM 1648a were purchased from the National Institute for Standards and Technology. CFA was purchased from Brandon Shores Unit power plant (Baltimore, MD). Particles were resuspended in PBS at 10 mg/ml and stored at −80°C until use. Working solutions were generated by diluting PM stocks in cell culture media. uPMs were not sterilized by autoclaving to preserve the entirety of the real-world exposure. PM characteristics are summarized in Table I.

**Table I.** Characteristics of the PM samples used in this study

PM Type (Source)	Collection System	Collection Date(s)	Size Range	Physical/Chemical Characterization
SRM 1648a (PM)	Collected in St. Louis, MO, in a purpose-built baghouse	Collected from 1976 to 1977	Bulk PM Mean diameter = 5.85 µm	13% carbon by mass, of which 10.5% is organic; high levels of metals (e.g., zinc, iron) and other inorganic elements
SRM 1650b (DEP)	Collected from four-cycle heavy-duty diesel engines; obtained from coordinating research council, Atlanta, GA	Collected in 1983	Bulk PM Mean diameter = 0.18 µm Size between 0.12 and 0.33 µm	Derived from SRM1650a
CFA	Brandon Shores Unit, Baltimore, MD	Collected in 1998	Size between 10 and 100 µm	The principal components of bituminous CFA are silica, alumina, iron oxide with varying amounts of carbon
Phoenix PM	Collected in Maricopa Co., AZ, using a high-volume sequential cyclone^*a*^	Collected summer (July) 2008	0.3 < d < 2.5 Size between 0.3 and 2.5 µm	Characterized for metals and ions Mean concentration = 9.33 ± 1.96 mg/m^3^
Houston PM	Collected in Harris Co, TX, using a high-volume sequential cyclone^*a*^	Collected summer (June) 2009	0.3 < d < 2.5 Size between 0.3 and 2.5 µm	Characterized for metals and ions Mean concentration = 8.68 ± 2.98 mg/m^3^
Pittsburgh PM	Collected in Allegheny Co., PA, using a high-volume sequential cyclone^*a*^	Collected summer (June) 2009	0.3 < d < 2.5 Size between 0.3 and 2.5 µm	Characterized for metals and ions Mean concentration = 10.32 ± 2.81 mg/m^3^
Baltimore PM_10_	Collected in Baltimore, MD, with a modified high-volume sequential cyclone^*b*^	Collected spring (May) 2012	0.3 < d < 10 Size between 0.3 and 10 µm	Characterized for metals and ions Mean concentration = 14.3 ± 7.5 mg/m^3^

d = diameter.

a PM collected with sequential cyclone as described by Rule et al. (51).

b Modified by eliminating the middle cyclone that would have collected PM between 2.5 and 10 μm.

### Sources of allergen

HDM and ragweed extracts (GreerStallergenes) were reconstituted in PBS and diluted to working solutions in cell culture media.

### Mice

C57BL/6 (strain 000664), *Nlrp3* knockout (KO; strain 021302), *Casp1/4* KO (strain 016621), *Tlr4* KO (strain 029015), and *Cd36* KO (strain 019006) mice were obtained from Jackson Labs. All mice were maintained in a specific pathogen-free facility and used according to the Institutional Animal Care and Use Committee.

### BMDM culture

Tibias and femurs from male C57BL/6J mice were removed, and marrow was harvested. Bone marrow cells were cultured on non–tissue culture–treated dishes and incubated in complete RPMI (cRPMI; 10% FBS, penicillin/streptomycin, l-glutamine, and 2-ME) supplemented with 15% L929-conditioned media for 7 d. BMDMs were washed once with PBS and harvested using Cell Stripper (Corning). For ELISAs, BMDMs were plated at 5 × 10^4^ cells/well of 96-well flat-bottom dish and left to adhere overnight in cRPMI. The following day, cells were exposed to particulates for 24 h, and supernatants were harvested for ELISAs. For other assays, cells were seeded at 2 × 10^6^ cells/dish in small non–tissue culture–treated dishes and left to adhere overnight in cRPMI. Cells were then exposed to particulates for 24 h and analyzed by flow cytometry.

### Chemicals

Inhibitors were purchased from Cayman Chemicals or Sigma.

### ELISA

Mouse IL-1α and TNF-α were detected using DuoSets (R&D Systems).

### Flow cytometry

BMDMs were stained for viability using the Zombie Aqua Fixable dye (BioLegend), followed by Fc blocking using 20 µg/ml anti-CD16/32 (clone 2.4G2; BioXCell) for 20 min. Cells were stained with BV421-conjugated anti-CD64 (clone X54-5/7.1; BioLegend) and acquired on a BD LSRII. Data were analyzed using FlowJo v10 (BD Biosciences).

### C12FDG-based detection of β-galactosidase activity

BMDMs were exposed to 100 nM bafilomycin A (Cayman Chemicals), then treated with 1 µM C12FDG (Cayman Chemicals) for 20 min at 37°C. Cells were washed and stained for surface markers, then analyzed by flow cytometry.

### Cell proliferation measured using the CellTiter-Glo assay

BMDMs were seeded at 1.0 × 10^4^ cells/well in a flat-bottom 96-well plate in cRPMI supplemented with 15% L929-conditioned media and stimulated as indicated. The CellTiter-Glo 2.0 assay (Promega) was then used to quantify ATP as a proxy for cell viability at 24, 48, and 72 h poststimulation.

### Statistical analysis

Data were analyzed via one-way ANOVA followed by Dunnett post hoc test for multiple comparisons. Significance was defined as *p* < 0.05. Analyses were executed using GraphPad Prism 9.

## Results

### Macrophages exposed to uPM acquire a senescent profile

**FIGURE 1.**
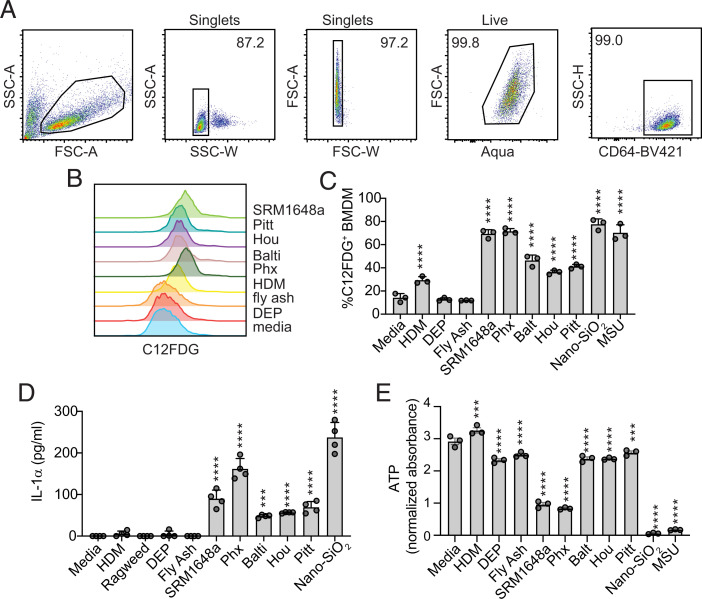
uPM drives senescence in macrophages. BMDMs were treated as indicated with media, sources of allergens (HDM and ragweed), or particulates (DEP, CFA, SRM 1648a, Phoenix [Phx], Baltimore [Balti], Houston [Hou], Pittsburgh [Pitt], nano-SiO_2_, or MSU crystals). Allergens were used at 50 µg/ml and particulates at 100 µg/ml. (**A**) Flow gating schema of CD64^+^ BMDMs. (**B**) Frequency of C12FDG^+^ BMDMs 24 h poststimulation. (**C**) Proliferation was measured using intracellular ATP 24 h poststimulation, normalized to baseline absorbance (0 h). (**D**) Supernatant levels of IL-1α were quantified by ELISA 24 h poststimulation. (**E**) Cell proliferation as measured using an ATP assay of BMDMs exposed to particulates and allergens as indicated for 72 h. Data plotted are technical replicates and are representative of at least two independent experiments. Data were analyzed using one-way ANOVA followed by Dunnett test for multiple comparisons. ***p <  0.001, ****p < 0.0001 versus media control.

We first wanted to determine whether uPM could drive manifestations of senescence in macrophages (Table I). To generate macrophages, we cultured bone marrow in differentiation media, yielding a pure macrophage culture, as determined by flow cytometry (Fig. 1A). One of the major determinants of this phenotype is the accumulation of senescence-associated β-galactosidase (β-gal; SA-β-gal), a consequence of increased lysosomal mass. Cells were pretreated with the vacuolar ATPase BafA1 to increase lysosomal pH, because SA-β-gal is often distinguished by its ability to function at a pH of up to 6 (versus 4 for “normal” β-gal, although there is evidence of variation between cell types for β-gal activity) (25). To assess for SA-β-gal activity, we used C12FDG, a fluorescent substrate for β-gal. We found that PMs from various urban sources and nano-SiO_2_ significantly increased SA-β-gal activity (Fig. 1B, 1C). This effect was not observed for particulates such as DEP or CFA, but only mildly for non-PM environmental exposures such as HDM.

In addition to SA-β-gal, senescent cells can adopt a specific secretome called the senescence-associated secretory phenotype (SASP). The SASP is characterized by the secretion of particular mediators, notably the cytokine IL-1α (26). IL-1α production is a salient characteristic of particulate exposure in macrophages and is a driver of particulate-induced inflammation (23). BMDMs were exposed to various particulates, including nano-SiO_2_, DEP, and uPM, and two common sources of allergens, HDM and ragweed. Consistent with what we found for SA-β-gal, IL-1α secretion was induced by uPMs and nano-SiO_2_, but not by DEP, CFA, or allergens (Fig. 1D).

Finally, because senescent cells no longer divide, cell-cycle arrest is another key manifestation of senescence (18). Quantification of ATP levels was used as a proxy for cell proliferation. Compared with the media group, BMDMs exposed to HDM demonstrated slightly increased ATP levels over 72 h, indicating increased proliferation (Fig. 1E). Conversely, cells exposed to PMs showed decreased proliferation (Fig. 1E). Our data suggest that exposure to uPM, but not allergen, promotes macrophage senescence.

### Senolytics abrogate senescence in uPM-treated BMDMs

**FIGURE 2.**
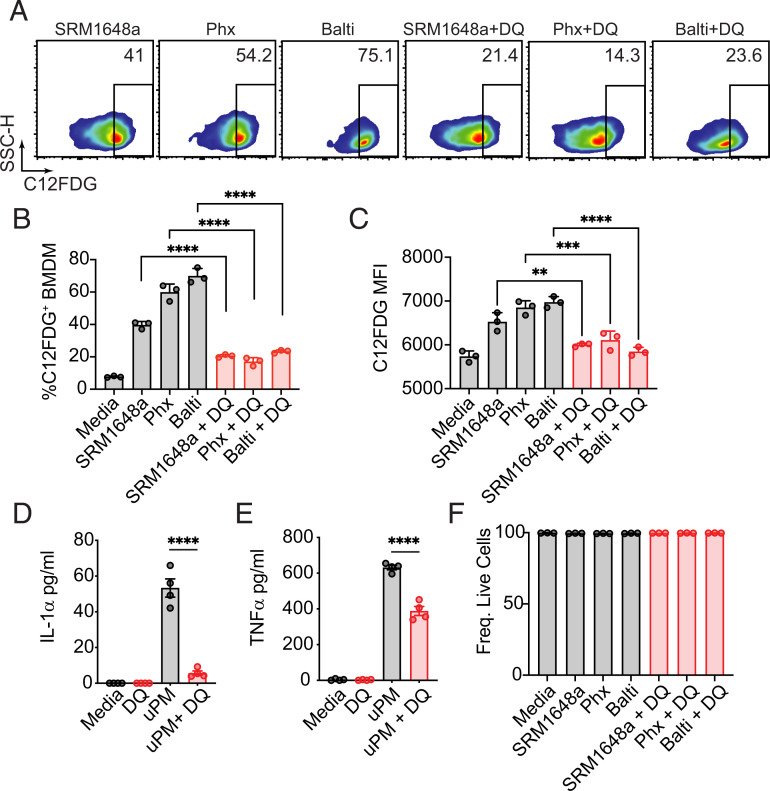
Senolytics abrogate uPM-induced senescence. BMDMs were treated with vehicle control or a mixture of D (0.3 µM) and Q (30 µM) in combination with 100 µg/ml urban particulates as indicated. (**A**) Representative flow plots showing frequency of C12FDG^+^ cells 24 h postsimulation, with corresponding (**B**) frequency of C12FDG^+^ cells and (**C**) median fluorescent intensity of C12FDG. Supernatant levels of (**D**) IL-1α and (**E**) TNF-α were quantified by ELISA after stimulation with SRM 1648a (100 µg/ml, 24 h). (**F**) Senolytic treatment does not induce cell death. Data plotted are technical replicates and are representative of at least two independent experiments. Data were analyzed using one-way ANOVA followed by Dunnett test for multiple comparisons. **p < 0.01, ***p < 0.001, ****p < 0.0001.

Senolytics are a drug class identified for their ability to eliminate senescent cells, and these include the Food and Drug Administration–approved tyrosine kinase inhibitor dasatinib (D) and the flavonoid quercetin (Q) (27). We found that DQ-treated BMDMs showed a significant reduction in uPM-induced SA-β-gal activity (Fig. 2A–C), as measured by frequency of SA-β-gal^+^ BMDMs (Fig. 2B) and median fluorescence intensity of SA-β-gal in BMDMs (Fig. 2C). DQ also abrogated uPM-induced IL-1α (Fig. 2D) but had only a partial effect on uPM-induced TNF-α (Fig. 2E). DQ-induced reduction in senescence was not due to enhanced mortality of senescent macrophages (Fig. 2F). Thus, DQ induced a reversal in uPM-induced senescence.

### uPM-induced IL-1α release is independent of the inflammasome, TLR4, scavenger receptors, and NADPH oxidase–derived reactive oxygen species

**FIGURE 3.**
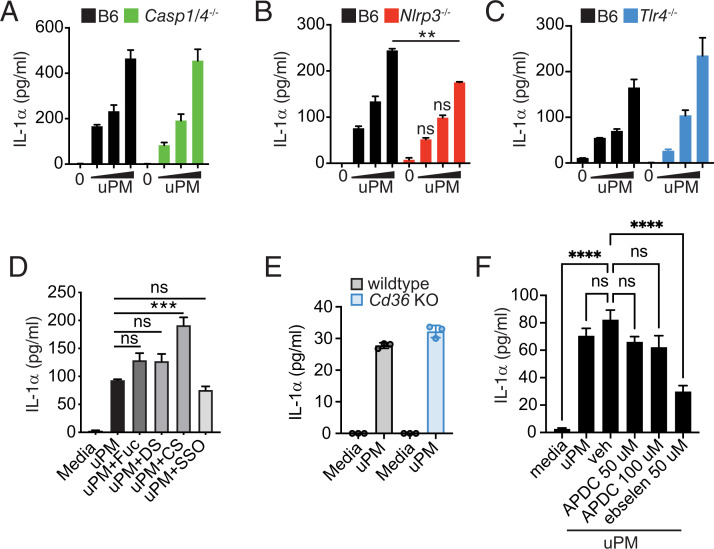
uPM-induced SASP is independent of caspase-1, NLPR3, TLR4, and SRs. Levels of supernatant IL-1α after uPM stimulation (SRM 1648a; 25, 50, and 100 µg/ml; 24 h) of (**A**) B6 and *Casp1/4*^−/−^ BMDMs, (**B**) B6 and *Nlrp3^−/−^*, and (**C**) B6 and *Tlr4^−/−^* BMDMs. (**D**) Supernatant IL-1α levels from BMDMs pretreated with SR inhibitors before stimulation with uPM (SRM 1648a, 100 µg/ml, 24 h). SR inhibitors fucoidan (Fuc; 100 µg/ml), dextran sulfate (DS; 100 µg/ml), and its control chondroitin sulfate (CS; 100 µg/ml) were used, as well as the CD36 inhibitor, sulfo-*N*-succidinimyl oleate (SSO; 200 µM). (**E**) Supernatant IL-1α levels from B6 and *Cd36*^−/−^ BMDMs stimulated with uPM (50 µg/ml). (**F**) Supernatant IL-1α levels from B6 BMDMs stimulated with uPM (50 µg/ml) in combination with vehicle (0.1% DMSO), APDC, or ebselen. Data are plotted as mean + SEM and are representative of at least two independent experiments. Data were analyzed using one-way ANOVA followed by Dunnett test for multiple comparisons. **p < 0.01, ***p <  0.001, ****p < 0.0001.

The inflammasome has been linked to the response of macrophages to PM (28). Thus, we wanted to ascertain the role of this pathway in mediating the adoption of the SASP in response to uPM. BMDMs from *Casp1/4*^−/−^, *Nlrp3^−/−^*, and *Tlr4^−/−^* mice were evaluated for IL-1α secretion to ascertain the relevance of the inflammasome and endotoxin-sensing pathways in uPM-mediated SASP. All inflammasome activators are thought to induce both IL-1α and IL-1β. These activators can be categorized as nonparticulate (LPS, ATP, nigericin) or particulate (MSU, alum, nano-SiO_2_, uPM). Whereas nonparticulate activators depend entirely on the inflammasome and caspase-1 for IL-1α secretion (29, 30), we report that uPM exposure drives IL-1α independently of caspase-1/4, NLRP3, and TLR4 activation (Fig. 3A–C). This is consistent with a previous report showing that silica- and MSU-induced IL-1α release is partially independent of inflammasome signaling (30).

Some groups have shown that scavenger receptors (SRs) can interact with particulates, including silica, DEP, and titanium oxide particles (22, 24, 31, 32). Although such particulates do not recapitulate the complexity of uPM, we wanted to test the contribution of SRs to the response observed on uPM exposure. We pretreated BMDMs with broad inhibitors of SRs, which inhibit both class A and B receptors, then exposed cells to uPM. We found that IL-1α release was not dependent on SRs (Fig. 3D). Moreover, genetic KO of the SR, CD36, failed to block uPM-induced IL-1α production (Fig. 3E). These data suggest that SR signaling is not required for induction of the SASP phenotype in BMDMs. Given the complex composition of uPM, it is likely that multiple mechanisms are involved in PM sensing.

Reactive oxygen species (ROS) are an important marker of cellular stress and can potentiate the transition of a cell to a senescent phenotype (33). To determine whether ROS plays a role in uPM-induced senescence, we treated BMDMs with the antioxidants ebselen and ammonium pyrrolidinedithiocarbamic acid (APDC). We found that blockade of NADPH oxidase–induced ROS via APDC did not prevent uPM-induced IL-1α release (Fig. 3F). However, scavenging of the oxidant peroxynitrite using ebselen partially reduced uPM-induced IL-1α. Overall, our data suggest that typical macrophage activation pathways do not drive uPM-induced SASP.

### uPM-induced macrophage senescence requires intact phagolysosome function

**FIGURE 4.**
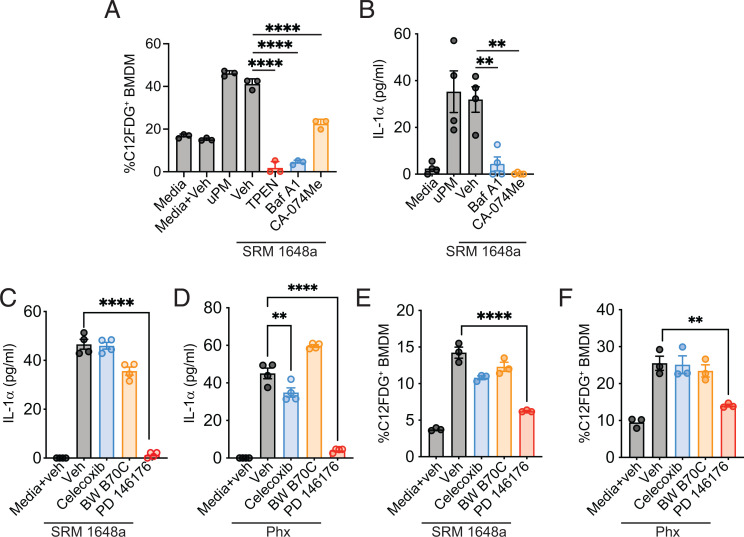
uPM-induced markers of senescence are dependent on a phagolysosome–15-LO pathway. (**A**) Frequency of C12FDG^+^ BMDMs was analyzed in uPM-stimulated cells (100 µg/ml SRM 1648a, 24 h) concurrently exposed to phagolysosome inhibitors: zinc chelator TPEN (20 µM), vacuolar ATPase inhibitor bafilomycin A1 (BafA1; 40 nM), and cathepsin B inhibitor CA-074Me (10 µM). (**B**) Supernatant IL-1α levels from BMDMs stimulated with uPM (100 µg/ml SRM 1648a, 24 h) and phagolysosome inhibitors BafA1 (40 nM) and CA-074-Me (10 µM). Supernatant IL-1α levels from BMDMs stimulated with 100 μg/ml (**C**) SRM 1648a or (**D**) Phoenix (Phx)-uPM in the presence of vehicle control and/or inhibitors of lipid pathway enzymes, celecoxib (10 µM), BW B70C (10 µM), and PD 176146 (10 µM) for 24 h. Frequency of C12FDG^+^ BMDMs stimulated with 100 μg/ml (**E**) SRM 1648a or (**F**) Phx-uPM in combination with vehicle control or inhibitors as indicated for 24 h. Data are plotted as mean + SEM and are representative of at least two to three independent experiments. Data were analyzed using one-way ANOVA followed by Dunnett test for multiple comparisons. **p < 0.01, ****p < 0.0001.

We next examined other pathways that may regulate uPM-induced markers of senescence. Because the formation of phagolysosomes is a critical step after phagocytosis, we sought to assess the importance of this pathway in uPM-induced senescence by using inhibitors targeting the phagolysosome pathway. First, the zinc chelator TPEN was used to disrupt lysosome function (34). In addition, phagolysosome maturation was inhibited by blocking acidification of the luminal pH with BafA1, a vacuolar H^+^-ATPase inhibitor. Finally, the cathepsin B inhibitor CA-074Me was used to block proteolysis and prevent optimal lysosome function and integrity (35). Blockade of these three pathways led to a complete reversal of uPM-induced SA-β-gal (Fig. 4A).

Next, we examined whether an intact phagolysosome pathway also affected uPM-induced SASP. Like with SA-β-gal, BafA1-mediated lysosome basification and cathepsin B inhibition completely inhibited uPM-elicited IL-1α (Fig. 4B). Lysosomes also have a role in lipid metabolism, and senescent cells often accumulate fatty acids, a substrate for enzymes, including lipoxygenases (LOs) and cyclooxygenases (COXs) (36). To investigate this pathway, we treated cells with the 5-LO inhibitor BW B70C, the 15-LO inhibitor PD 146176, and the COX-2 inhibitor celecoxib. We found that inhibition of 15-LO, but not 5-LO or COX-2, ablated IL-1α production in response to uPM (Fig. 4C, 4D). Consistent with this, 15-LO also drove uPM-induced SA-β-gal accumulation (Fig. 4E, 4F). These data position 15-LO as a potentially important mediator of macrophage senescence.

**FIGURE 5.**
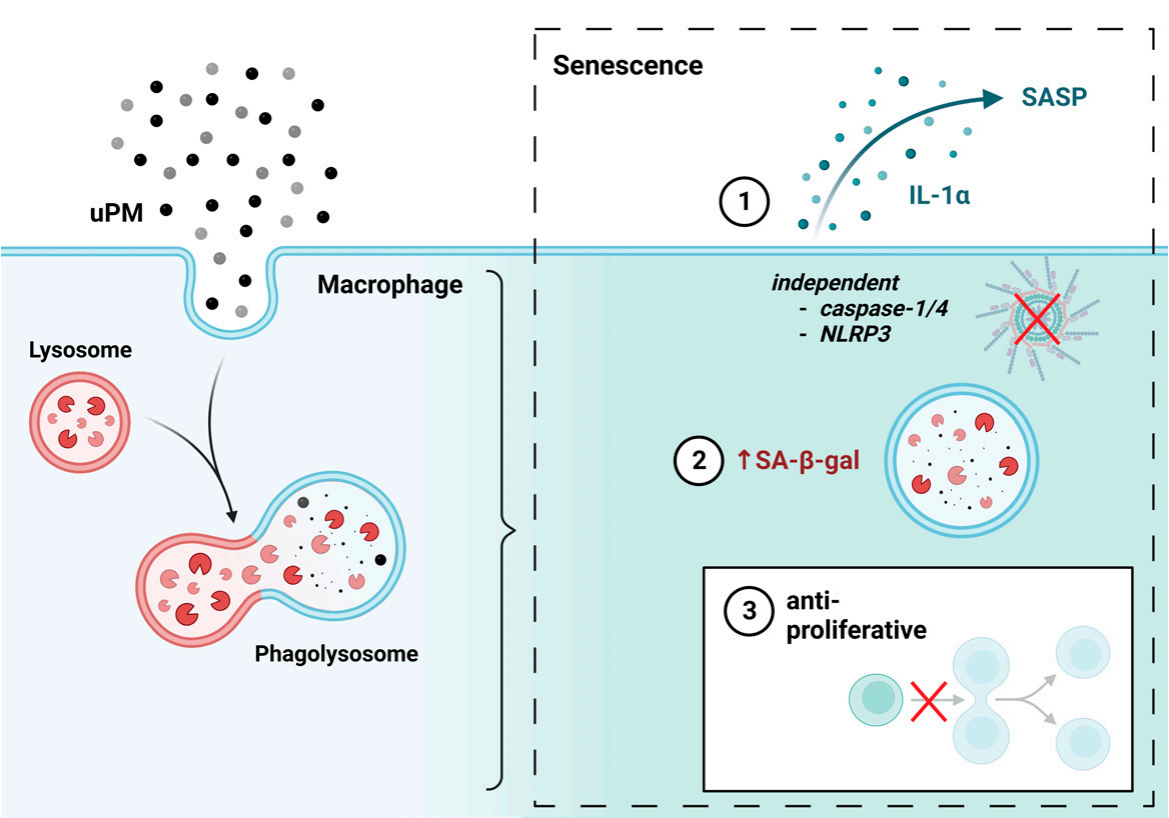
Phagolysosome processing of uPM triggers macrophage senescence. Macrophage phagocytosis and subsequent processing of uPM result in the acquisition of a cell profile indicative of senescence, as measured by SASP production (IL-1α), increased SA-β-gal activity, and inhibition of proliferation. This phenotypic shift is independent of caspase-1/4 and NLRP3. The figure was created using BioRender.com.

Collectively, we propose that a phagolysosome processing of uPM and further activation of 15-LO serves as a triggering event for macrophage senescence, as measured by SASP adoption, SA-β-gal activity, and cell-cycle arrest (Fig. 5).

## Discussion

Air pollution remains a significant public health challenge in both scope and severity, adversely affecting millions worldwide. Despite investigation into the health implications of PM exposure being in its nascent stages temporally, PM has been associated with myriad consequences to human health spanning diverse systems, including the immune system (5, 8, 9). Although PM has been proposed as a driver of dysregulated immunity, the mechanisms that give rise to dysfunction remain to be fully elucidated.

As critical sentinels in the lung, macrophages are among the first cell types to encounter airborne insults and are critical in coordinating the subsequent immune response (10, 37). PM-induced senescence has been reported in the context of some cell types, including fibroblasts and epithelial cells (11–13, 38), but not macrophages. Our data demonstrate that exposure of macrophages to uPM results in senescence. These changes are uniquely observed in response to uPM, but not other commonly used sources of PM (such as DEP or CFA) or other environmental triggers, such as allergens.

Senolytics are a recent pharmacological tool for selective targeting of senescent cells. Murine studies into the therapeutic potential of DQ have demonstrated a reduction in senescent cells and improved functionality in myriad contexts, including diet-induced obesity, renal fibrosis, and osteoporosis (39). In line with these data, we found that the manifestations of senescence induced in macrophages by uPM could be reversed by senolytics.

The relationship between inflammation and senescence is complex and likely bidirectional (40). Blocking canonical pathways of macrophage activation, such as the inflammasome, caspase-1/4, TLR4, SRs, and ROS, failed to prevent uPM induction of SASP. This is in contrast with a report that caspase-4 drives LPS-induced SA-β-gal and cell-cycle arrest in human fibroblasts (41), supporting a distinction between the mechanisms by which classical inflammatory triggers (e.g., LPS) and uPM drive senescence. Moreover, we found that phagolysosome activity drives uPM-induced senescence, suggesting phagolysosome processing of uPM by macrophages is key to the development of cellular senescence. Although our data demonstrate that uPM-induced SASP is TLR4 independent, it should be noted that intracellular LPS can be sensed in a TLR4-independent manner, via CD14-dependent cytosolic sensing (42–44).

Senescence is associated with cytosolic accumulation of fatty acids, which are released by the lysosome and metabolized by various enzymes, including COX-2, 5-LO, and 15-LO. 15-LO is a known tumor suppressor linked to cellular senescence in prostate epithelial cells, as measured by SA-β-gal induction and cell-cycle arrest (45). In line with this, the blockade of 15-LO function abrogates uPM-induced SASP. Interestingly, inhibition of 15-LO has been shown to decrease gene expression of inducible NO synthase, which produces peroxynitrite (46). This may explain why ebselen scavenging of peroxynitrite partially reduced IL-1α production in uPM-exposed BMDMs.

Although our findings offer insight into the role of uPM as a driver of macrophage senescence, BMDMs are ontologically akin to interstitial macrophages and less so to yolk sac–derived alveolar macrophages. Thus, they may not recapitulate the phenotype of all pulmonary macrophages. Although there are few data describing uPM-induced senescence in human cells, there is precedent for uPM-mediated immune dysfunction in humans. For example, PM exposure has been demonstrated to induce senescence in human keratinocytes (47) and elicit TNF-α production in human alveolar macrophages (48), consistent with our findings in BMDMs. Although the range of uPM concentrations we have tested (25, 50, and 100 µg/ml) all induced manifestations of senescence, we have not identified the minimal dose of uPM that could drive this effect, although lower concentrations (1 and 5 μg/ml) have been reported to induce dysfunction in *Mycobacterium tuberculosis*–exposed human PBMCs (49, 50). In vitro uPM concentrations are difficult to translate to real-world exposures; thus, the real-world translatability of our results is limited by our in vitro approach. Nevertheless, our data provide preliminary justification for the future interrogation of uPM-induced macrophage senescence in vivo in mouse models and in human pulmonary macrophages. Thus, although our studies focused on mechanistic pathways downstream of uPM exposure that drive cellular senescence, future studies investigating this biology in vivo are warranted.

Exposure to uPM may diminish macrophage functionality by driving senescence, thereby contributing to impaired tissue function. We posit that uPM-induced macrophage senescence may be a key facet of the pathophysiology driving uPM-induced immune dysfunction. Therefore, our data warrant further exploration of this phenomenon in vivo and in human pulmonary macrophages.
